# Gastric Ectopic Pancreas: A Pediatric Case Report

**DOI:** 10.7759/cureus.107937

**Published:** 2026-04-29

**Authors:** Imane Chemlal, Amal Hamami, Maria Rkain

**Affiliations:** 1 Department of Pediatrics, Mohammed VI University Hospital, Oujda, MAR

**Keywords:** gastric ectopic pancreas, incidental finding, pediatric esophagogastroduodenoscopy, submucosal gastric lesion, superficial biopsy

## Abstract

An ectopic pancreas is a congenital anomaly in which pancreatic glandular tissue is found outside its normal anatomical location. Specifically, while it can occur anywhere along the gastrointestinal tract, its presence in the stomach is relatively rare in children and is often asymptomatic, typically discovered incidentally.

Our case involves a six-year-old child who underwent an esophagogastroduodenoscopy due to the suspicion of celiac disease in the context of iron-deficiency anemia that did not respond to oral iron therapy, despite the absence of associated clinical symptoms and a normal physical examination. The esophagogastroduodenoscopy incidentally revealed a well-defined, 5-mm submucosal nodular lesion in the prepyloric region, with a central depression, characteristic of an ectopic gastric pancreas. Histological examination of the duodenal biopsies confirmed coeliac disease. In contrast, the biopsy of the lesion showed no evidence of neoplasia and was considered normal.

Our case highlights the diagnostic challenge posed by normal histology in the context of suspected gastric ectopic pancreas, as superficial biopsies may be non-contributory due to its submucosal location. It also underscores the importance of recognizing the typical endoscopic features to avoid misdiagnosis and unnecessary invasive procedures in pediatric patients.

## Introduction

Gastric ectopic pancreas is a rare congenital anomaly in children, with a prevalence of 0.54% to 1.1% [1,2]. Although the exact embryological origin remains unclear, the dislocation hypothesis is the most widely accepted [3]. This theory is based on mechanical disturbances occurring between the fourth and seventh weeks of gestation [4]. The pancreas derives from the endoderm of the primitive gut and develops from two separate embryonic primordia, the ventral and dorsal buds, which appear as early as the fourth week of gestation and undergo rapid growth and significant morphologic reorganization from the fifth week onward [4]. Concurrently, the rightward rotation of the duodenum drives the posterior migration of the ventral bud around the duodenum until it fuses with the dorsal bud between the sixth and seventh weeks. During these complex processes, fragments of pancreatic tissue may detach from the main pancreas and implant in other locations, most commonly within the gastrointestinal tract [3,4]. This lesion is usually benign and remains stable over several years of follow-up. In very rare cases, it may undergo malignant transformation [1,2].

Most cases are asymptomatic and are discovered incidentally during an esophagogastroduodenoscopy or surgical procedures performed for unrelated indications. In rare cases, they may present with nonspecific clinical signs related to complications of ectopic pancreas, such as abdominal pain, vomiting, gastrointestinal bleeding, dyspepsia, or bowel obstruction secondary to obstruction, volvulus, or intussusception [2,3].

Endoscopically, gastric ectopic pancreas exhibits a typical appearance: a small submucosal nodule, generally measuring less than 2 cm in diameter [1], often with a central umbilication, which can help distinguish it from other submucosal gastric lesions [2]. Although histological examination is considered the gold standard, a normal biopsy does not exclude the diagnosis of this lesion if other diagnoses have been ruled out. This is largely due to the superficial nature of standard endoscopic biopsies, which often fail to reach the submucosal pancreatic tissue [1,5].

We present a case of ectopic gastric pancreas with typical endoscopic features observed during oesophagogastroduodenoscopy. This case describes the diagnostic challenge of this lesion when superficial biopsies are non-diagnostic, illustrating a well-documented diagnostic pitfall in the literature. Identifying these typical endoscopic features enables diagnosis, supports appropriate management, and helps avoid unnecessary procedures in pediatric patients.

## Case presentation

We present the case of a six-year-old boy with a history of surgically corrected bilateral postaxial polydactyly, who was referred to our department for evaluation of iron-deficiency anemia refractory to oral iron therapy. The anemia was discovered incidentally at the age of two during a preoperative laboratory workup for circumcision. There was no improvement despite iron supplementation.

On admission to our pediatric gastroenterology department, physical examination revealed normal vital signs, normal temperature, mild conjunctival pallor, a height of 122 cm (+1 standard deviation), and a weight of 20 kg (within normal range). No other clinical abnormalities were observed.

Initial investigations revealed hypochromic, microcytic anemia (hemoglobin: 7.4 g/dL; mean corpuscular volume (MCV): 58.3 fL; mean corpuscular hemoglobin concentration (MCHC): 27 g/dL), associated with markedly low ferritin levels (3.15 ng/mL). The rest of the laboratory workup, including anti-tissue transglutaminase IgA antibodies, was positive, raising suspicion of celiac disease. However, the child remained asymptomatic. The detailed laboratory results are presented in Table 1.

**Table 1:** Laboratory findings of the patient. Hb: hemoglobin; Hct: hematocrit; MCV: mean corpuscular volume; MCH: mean corpuscular hemoglobin; MCHC: mean corpuscular hemoglobin concentration; PLT: platelets; WBC: white blood cell count; ANC: absolute neutrophil count

Parameters	Result	Reference Range
Hb	7.4 g/dL	13-18 g/dL
Hct	27.4%	40-54%
MCV	58.30 fL	80.00-98.00 fL
MCH	15.70 pg	27.00-32.00 pg
MCHC	27.00%	32.00-36.00%
PLT	222,000 cells/µL	150,000-400,000 cells/µL
WBC	11,050 cells/µL	4,000 -10,000 cells/µL
ANC	6,220 cells/µL	1,500-7,000 cells/µL
Lymphocytes	3,570 cells/µL	1,000-4,000 cells/µL
Monocytes	930 cells/µL	200-800 cells/µL
Eosinophils	280 cells/µL	0-500 cells/µL
Basophils	50 cells/µL	0-200 /µL
Ferritin	3.15 ng/mL	22-275 ng/mL
Anti-transglutaminase IgA	21 IU/mL	< 10 IU/mL

**Figure 1:**
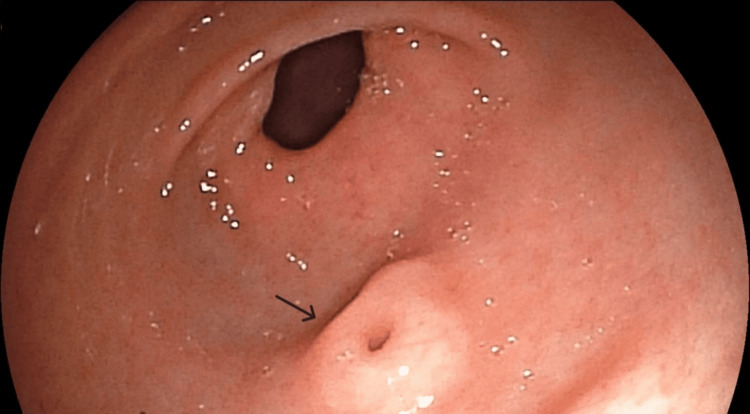
A 5-mm umbilicated prepyloric submucosal nodule (arrow), located along the greater curvature of the gastric antrum, is typical of a gastric ectopic pancreas.

**Figure 2:**
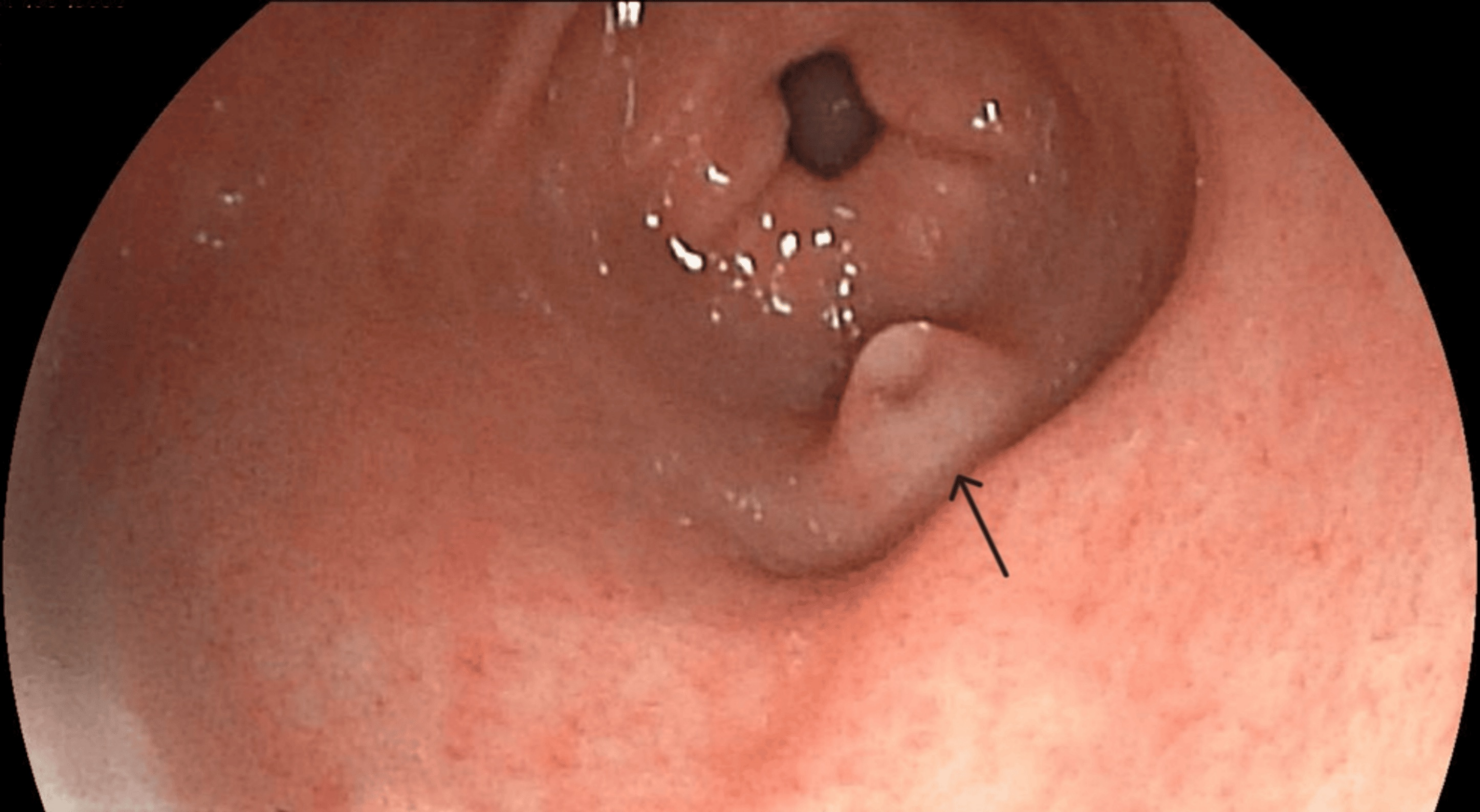
Typical endoscopic appearance of a gastric ectopic pancreas (arrow) in this case.

An esophagogastroduodenoscopy revealed moderate duodenal atrophy and, incidentally, identified a 5-mm prepyloric submucosal nodule with regular margins and a central umbilication. The nodule was located along the greater curvature of the gastric antrum (Figures 1-2). 

**Figure 3:**
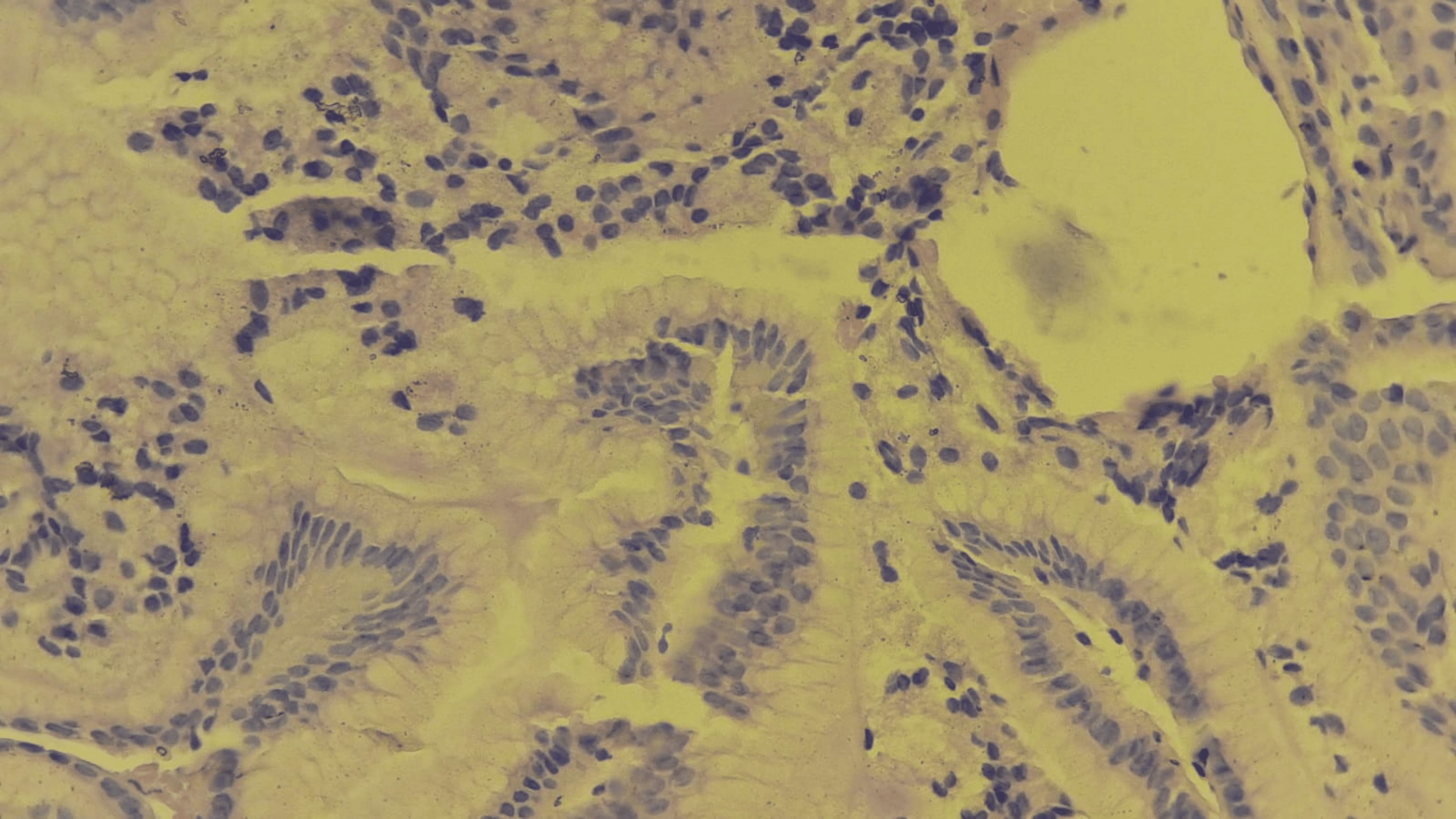
Histopathological examination of the lesion. Histopathological examination of the lesion (H&E ×40) reveals gastric mucosa with a mucinous surface epithelium and a closed apical pole, overlying a superficial portion of the chorion, which contains mononuclear inflammatory cells, without other abnormalities. H&E, hematoxylin and eosin

Histological examination of the gastric lesion showed normal mucosa with no evidence of malignancy or other anomalies (Figure 3). In contrast, duodenal biopsies confirmed the diagnosis of celiac disease, which likely explains the iron-deficiency anemia. Abdominal magnetic resonance imaging (MRI) was unremarkable.

In the absence of pancreatic tissue on histological examination and, given the lack of evidence of malignancy or other abnormalities, the diagnosis of gastric ectopic pancreas was considered based on characteristic endoscopic features, including its typical antral location, submucosal appearance, and central umbilication, as described in the literature.

Given the absence of symptoms and the lesion’s benign endoscopic and histological features, a conservative management approach was adopted. This approach involves regular clinical and endoscopic follow-up to ensure lesion stability and detect any potential complications. The child was also placed on a gluten-free diet following the diagnosis of celiac disease. 

## Discussion

Ectopic pancreas, also known as aberrant pancreas, is characterized by the presence of pancreatic tissue in an ectopic location without structural, neural, or vascular connections to the normal pancreas [6]. While its exact embryological origin is uncertain, the dislocation theory is the most widely accepted [3].

This rare congenital malformation is predominantly seen in children aged ≤1 year (46.6%) and is more prevalent in boys [7]. In the pediatric population, its prevalence is low: 0.54% among patients who underwent bowel exploration and 1.1% among children without a history of esophageal atresia or trisomy 21 who underwent esophagogastroduodenoscopy for other reasons [1,2]. This is consistent with our study, in which only one case was identified among 362 esophagogastroduodenoscopies, corresponding to a prevalence of 0.27%. This finding highlights the extreme rarity of this malformation in the pediatric population.

The lesion can occur anywhere in the gastrointestinal tract, most frequently in Meckel's diverticulum (46.59%) and the small intestine. Gastric involvement is less common (6.82%) [7]. As observed in our case, gastric ectopic pancreas most commonly occurs in the gastric antrum as a submucosal nodule. It is frequently found along the greater curvature, typically within 50 mm of the pylorus. This is consistent with the majority of cases reported in the literature [1,7].

Most cases (75%) are asymptomatic and detected fortuitously during endoscopic or surgical procedures performed for other indications [1,7]. However, some patients may develop symptoms secondary to complications such as inflammation, bleeding, intestinal obstruction, or perforation. These complications are most frequently observed after the age of five (53.33%) [7]. When present, these symptoms are generally nonspecific, and their severity and clinical presentation depend on the lesion's anatomical location and size [8]. Although rare, studies have described symptomatic ectopic pancreas, as reported by Oshiba et al., in which a two-month-old infant presented with persistent non-bilious vomiting secondary to an ectopic pancreas compressing the prepyloric region. This was treated surgically [9]. On the other hand, Cao et al. described the case of a 17-year-old child who presented with intermittent abdominal pain and melena secondary to ileal obstruction caused by an ectopic pancreas measuring 5 × 2 cm. This was also treated surgically [10]. These cases confirm that all symptoms are related to complications of this lesion.

A gastric heterotopic pancreas can be identified during esophagogastroduodenoscopy and presents with characteristic endoscopic features. As Seddon and Stringer noted, this can be done without histological confirmation. This condition typically presents as an isolated, round or oval submucosal lesion with well-defined borders and a broad base of implantation. It commonly ranges from 1 to 2 cm in size. The lesion predominantly arises in the antral region of the stomach. On cross-section, the lesion appears yellowish or whitish, and it may exhibit a central depression suggesting a ductal opening. However, the study also highlighted significant variation in the endoscopic appearance of these lesions [1]. 

Histological confirmation can be challenging because biopsies often show normal gastric mucosa due to the lesion's submucosal location. For instance, Archid et al.'s study found that only one of five cases was confirmed preoperatively [11]. This shows that normal biopsies do not exclude the diagnosis and result from superficial sampling [12]. This challenge was also observed in our case, where the biopsy did not reveal pancreatic tissue or, in particular, any tumor cells or other abnormalities. This highlights the importance of interpreting histological findings in conjunction with endoscopic features while systematically excluding other differential diagnoses, including neoplastic processes, rather than considering them in isolation. In this context, the characteristic endoscopic appearance remains key for diagnosis. This approach is supported by the literature. Pouessel et al. reported the incidental diagnosis of gastric ectopic pancreas in two children (aged 21 months and 2 years) based solely on typical endoscopic findings [13]. Similarly, Rubel and Chong described a 19-year-old patient in whom the diagnosis was established exclusively on endoscopic morphology [14]. Collectively, these reports, including our own case, highlight that in selected cases with typical features and no suspicion of malignancy, the diagnosis of gastric ectopic pancreas may be suggested based on endoscopic findings.

Nevertheless, this entity can mimic other submucosal lesions, such as lipomas, gastric carcinoma, or gastric lymphoma, as well as mesenchymal tumors, particularly gastrointestinal stromal tumors (GISTs), which are potentially malignant neoplasms most often located in the gastric body [15]. In this context, imaging techniques, particularly endoscopic ultrasound (EUS), computed tomography (CT), and MRI, play a key role in detecting ectopic pancreas and in differentiating it from other submucosal lesions. EUS enables precise localization of the lesion within the layers of the gastrointestinal wall, as well as assessment of its size and echogenicity. Typically, an ectopic pancreas appears as a submucosal lesion with a central umbilication, a long-to-short diameter ratio greater than one, indistinct margins, and mixed echogenicity [16]. In such cases, histological confirmation via invasive procedures is usually unnecessary [16]. In contrast, when the lesion is atypical, involves the deep muscular layer, and appears homogeneous or hypoechoic, other differential diagnoses should be considered, including mesenchymal tumors such as GIST, leiomyoma, schwannoma, or inflammatory fibroid polyp [16]. The combination of EUS and CT significantly improves differentiation from GISTs, potentially avoiding unnecessary surgical interventions. CT and MRI are more likely to detect ectopic pancreas when the lesion is sufficiently large. On contrast-enhanced CT, the lesion shows enhancement similar to that of normal pancreatic tissue, whereas on MRI it appears hyperintense on T1-weighted images and exhibits early arterial enhancement [17-19].

Management is guided by the clinical presentation. Conservative management is recommended for asymptomatic lesions with typical endoscopic features. However, if the lesion is clearly responsible for clinical symptoms, removal, either surgical or endoscopic, is indicated [1].

According to published series and reported cases in the literature, the majority of patients managed conservatively do not show lesion progression, complications, or symptom onset during follow-up. This favorable outcome is also illustrated by the case reported by Rubel and Chong, in which an ectopic pancreas, initially masked by a gastric hematoma secondary to an upper gastrointestinal hemorrhage, was discovered during a follow-up endoscopy performed after medical treatment and *Helicobacter pylori* eradication. The resolution of the hematoma allowed the underlying lesion to be identified without recurrence of hematemesis [14]. Similarly, the study by Seddon and Stringer describes incidentally discovered, asymptomatic cases that did not develop any complications attributable to this anomaly. In four children followed by repeated upper gastrointestinal endoscopies over a period of up to six years, no morphological change in the lesion was observed [1].

The main value of this case lies not only in its rarity but also in the diagnostic challenge it illustrates. It highlights the clinically silent nature of gastric ectopic pancreas and the potential discordance between highly suggestive endoscopic findings and normal superficial biopsies that lack pancreatic tissue. In our case, abdominal MRI was normal, likely because of the lesion's small size, and EUS was not available at our institution. Therefore, this diagnosis may be considered after excluding other differential diagnoses based on the typical endoscopic features described in the literature. In addition, the incidental and indolent discovery of the lesion in our patient, together with regular follow-up, allowed for the early detection of potential complications during ongoing surveillance.

## Conclusions

Gastric ectopic pancreas is a rare anomaly in children, with a prevalence of 0.27% in our department, and is most often asymptomatic. This lesion may resemble other gastric submucosal lesions, particularly those of tumoral origin, which justifies performing a biopsy to exclude neoplasia and other differential diagnoses. However, the absence of pancreatic tissue in the biopsy is due to the lesion's submucosal location. In this context, we can rely on the typical macroscopic endoscopic features to guide diagnosis and avoid unnecessary invasive investigations or surgical procedures. Our case illustrates the diagnostic challenge posed by this lesion due to its often silent presentation and the limitations of superficial biopsies, and underlines the importance of recognizing its typical endoscopic features to optimize patient management.
